# Editorial: Adaptive evolution of grasses

**DOI:** 10.3389/fpls.2023.1105320

**Published:** 2023-01-30

**Authors:** Xixi Liu, Xiaoyu Weng, Joseph Edwards, Lingqiang Wang, Chao Zhang, Jiehua Qiu, Zhiyong Li

**Affiliations:** ^1^ State Key Lab of Rice Biology, China National Rice Research Institute, Hangzhou, China; ^2^ Department of Integrative Biology, University of Texas at Austin, Austin, TX, United States; ^3^ State Key Laborary for Conservation & Utilization of Subtropical Agro-Bioresources, College of Agriculture, Guangxi University, Nanning, China; ^4^ State Key Laboratory of Crop Stress Biology for Arid Areas, College of Agronomy, Northwest A&F University, Yangling, China

**Keywords:** grasses, Poaceae, adaptive evolution, genomics, molecular breeding

The grass family, Poaceae, the species-rich plant families, consists of *c*. 10,000 species including the most economically important plants of modern times, providing over one-half of all dietary energy and comprising about one-third of Earth’s vegetative cover (Group et al., 2001; Bouchenak-Khelladi et al., 2008). It has been suggested that the genomes of Poaceae have evolved at an elevated rate due to the selection imposed by changing environmental conditions and more recent breeding efforts (Edwards and Smith, 2010; Stromberg, 2011). In this special issue “*Adaptive Evolution of Grasses*” seven articles were published that explore genetic/genomic resources in Poaceae species, discussed the involvements of grass biology in different developmental processes or under various stress, covering outstanding advances in molecular genetic basis of adaptive and agronomic traits in diverse grass research fields. In this editorial, we summarized the main findings of these seven insightful works.

Owing to the booming population, deteriorating environments and degradating farmland, it is imperative to explore new wild grass resources to improve the adaptation of the agroecosystem, ensure sustainable livelihoods and food security (Grassini et al., 2013; Ray et al., 2013). As autotrophic organisms, plants convert light energy into chemical energy, primarily in the form of carbohydrate molecules such as sugar, through photosynthesis to fuel the organism activities (Gifford and Evans, 2003; Jansson et al., 2018). It has been clear that the improvement of photosynthetic efficiency can significantly increase the accumulation of plant assimilations products, and the yields (Zhu et al., 2010; Walker et al., 2016; Li et al., 2022). Shen et al. provided an exhaustive overview of current knowledge on the cultivation of high light efficiency plants, including the research progress and methods in improving the photosynthetic efficiency of plants, and especially highlighted the preliminary exploration in the design of C4 crops by means of genetic engineering (Matsuoka et al., 2001). Based on the future problems and difficulties faced by the cultivation of high light efficiency plants, Shen et al. proposed a strategy for improving breeding efficiency.

Rice is one of the major crops worldwide, feeding over half of the global population (Panda et al., 2021). Shen et al. briefly reviewed the heat signal transmission mechanism of plant and the genetic basis in rice heat-tolerant at present. It is expected that the research on plant heat tolerance will contribute to meeting future global warming. Early seedling vigor (ESV) directly reflects seedling establishment (Rao et al., 2007). Moreover, rice plants with high ESV always show high flooding-tolerant, high competitive advantage over weeds, as well as better nutrient uptake (Luo et al., 2007; Wang et al., 2010; Wang et al., 2021). Ma et al. identified a candidate gene *qSL2* by genome-wide association study (GWAS) using 302 international diverse rice accessions. In addition, Ma et al. found *qSL2* contributed 3.05% variation across in whole panel and 7.38% variation across the *indica* subpopulation. Haplotype and RNA-seq analysis between long seedling length (SL) accessions and short SL accessions suggested *LOC_Os02g17780* (*OsCPS1*) may be the candidate gene of *qSL2*, which participates in GA biosynthesis. The research article provides a novel major QTL (*qSL2*) for ESV, which shows promising potential for direct seeding.

Epigenetic modifications, which include DNA methylation, post-translational modification of histone protein, and smallRNA (siRNA) biogenesis, play a pivotal role in regulation of plant development and response to biotic/abiotic stresses (Mirouze and Paszkowski, 2011; Baulcombe and Dean, 2014; Schmid et al., 2018; Liang et al., 2020). Hexaploid wheat (*Triticum aestivum* L.), one of the major cereal crops accounting for about a quarter of global cereal production, is sensitive to biotic/abiotic stresses (Tack et al., 2015; Yadav et al., 2022).

However, the knowledge of epigenetic modifications is still limited in plants (Agarwal et al., 2020). Wang et al. performed a comprehensive analysis of *JmjC* genes which encode demethylases that are involved in histone demethylation. A total of 24 wheat *JmjC* genes were identified and reported to be conserved in A, B, and D subgenomes. Furthermore, *JmjC* genes were proposed to play significant role in improving tolerance to drought stress in wheat. Likewise, Lu et al. identified 9 *Fes1s* genes in hexaploid wheat which are key components of heat shock protein 70 system. In Arabidopsis and rice, *Fe1p* homologues are reported to be involved in abiotic stresses (Zhang et al., 2010; Fu et al., 2020; Qian et al., 2021). Lu et al. found that overexpression of *TaFes1A-5A* and *TaFes1A-5D* could not complement the thermotolerance defect in Arabidopsis thermosensitive *fes1a* mutant,but could accelerate seed germination under both normal and heat stress conditions. The results of all these works further expand our knowledge of epigenetic modifications in response to abiotic stress.

Herbicide tolerance in grasses is more crucial than that in other plants in the modern agriculture (Powles and Yu, 2010). Epigenetic modifications are also known to regulate gene expression in adaptation of weedy grass species to the herbicide stress (Lu et al., 2016; Pan et al., 2022). Sen et al. reviewed the potential contributions and current challenges of epigenetic mechanisms in adaptive responses of grass-weedy species to herbicidal stress.

Over the recent decades, plant breeding has greatly benefited by the high-throughput sequencing technologies. The transcriptome sequencing technology in plants plays more advantageous role in molecular breeding even in absence of a reference genome (Zhang et al., 2018; Guo et al., 2021; Shaw et al., 2021). Xiong et al. reported full-length transcriptome sequence of *K. melanthera* by single-molecule real-time sequencing technology. Consequently, a total of 42,433 SSR markers were identified and 21 SSR markers showed good cross-species transferability among 56 K*. melanthera* accessions. Haque et al. demonstrated that coastal genotypes show superior salinity-tolerance and ion homeostasis compared to inland populations of *Panicum hallii*. Haque et al. further identified several QTLs associated with salinity-tolerance, and several differentially expressed candidate genes are included various ion transporters by genome-wide transcriptome analysis. Nevertheless, a causal relationship between these observations requires further investigation. These studies pave the way to conduct further molecular breeding research in grass, and provide valuable information for future evolutionary and genetic studies in abiotic stress tolerance in grasses. Overall, the articles published in this special issue are excellent examples of the recent advances made in the genetic evolution and local adaptation in grasses. We thank all the authors for their contributions and critical assessment on the issues. We also thank the Assistant Editor Ms. Lily Wyatt for providing us with the opportunity to serve as the Guest Editor or Topic Coordinator of this special issue “*Adaptive Evolution of Grasses*.”

## Author contributions

All the authors participated in the editing of this Research Topic. XL and ZL wrote the draft, and all the other authors provided suggestive comments on the editorial. All authors contributed to the article and approved the submitted version
